# Less than subtotal parathyroidectomy in multiple endocrine neoplasia type 1: A case report and review of the literature

**DOI:** 10.1016/j.ijscr.2020.10.140

**Published:** 2020-11-10

**Authors:** Diani Kartini, Filipus Dasawala, Maria Francisca Ham

**Affiliations:** a Department of Surgery, Surgical Oncology Subdivision, Dr. Cipto Mangunkusumo General Hospital, Faculty of Medicine, Universitas Indonesia, Jakarta, 10430, Indonesia; b Department of Surgery, Faculty of Medicine, Universitas Indonesia, Jakarta, 10430, Indonesia; c Department of Anatomical Pathology, Faculty of Medicine, Universitas Indonesia, Jakarta, 10430, Indonesia

**Keywords:** Primary hyperparathyroidism (PHPT), Multiple endocrine neoplasia type 1 (MEN1), Parathyroidectomy, Case report

## Abstract

**Introduction:**

Multiple endocrine neoplasia type 1 (MEN1) is a rare syndrome with common presenting signs and symptoms secondary to primary hyperparathyroidism (PHPT), which is managed surgically. Traditionally, either subtotal parathyroidectomy (SPTX) or total parathyroidectomy with autologous transplantation (TPTX) is the approach of choice. However, recent studies showed comparable persistence and recurrence rate in a subset of MEN1 patients (two or more concordant preoperative imaging results) who underwent less than subtotal parathyroidectomy (LSPTX).

**Presentation of case:**

We report a case of patient with PHPT and delayed diagnosis of MEN1, who underwent LSPTX without intraoperative parathyroid hormone (IOPTH) measurement. The approach was chosen based on the preoperative imaging studies. Unfortunately, the PHPT persisted and the patient was reoperated. To further elucidate the issue, a systematic search of the literature was conducted on Cochrane library, PubMed, and Scopus; articles relevant to the case were reviewed. Results are conflicting results with most of the studies showed LSPTX is inferior compared to the other two approaches.

**Discussion:**

Therefore, given the current body of evidence, we consider that subtotal or total parathyroidectomy is still the preferred surgical approach for the treatment of PHPT in MEN1.

**Conclusion:**

Further studies are still needed to see whether LSPTX is comparable to SPTX or TPTX in regards to persistent and recurrent hyperparathyroidism if the conditions are met.

## Highlights

• Traditionally, the surgical approach for primary hyperparathyroidism in MEN1 is either subtotal or total parathyroidectomy.
• Advances in medical imaging allows more accurate preoperative localization of abnormal parathyroid gland.
• Recent studies showed less than subtotal parathyroidectomy has comparable outcome compared to subtotal or total parathyroidectomy.
• Further studies are needed to determine whether there are subsets of MEN1 patients that can benefit from less than subtotal parathyroidectomy.

## 1 Introduction

Multiple endocrine neoplasia type 1 (MEN1) is a rare autosomal dominant disorder causing endocrine tumors due to inactivating mutation of MEN1 gene [1]. Its prevalence is 2–3/100,000 with the most common clinical presentations relating to signs and symptoms of primary hyperparathyroidism (PHPT). Currently, subtotal parathyroidectomy (SPTX) or total parathyroidectomy followed by autotransplantation (TPTX) is the treatment of choice [2]. However, advances in medical imaging enables more accurate preoperative localization of abnormal parathyroid gland. Hence, the option of less than subtotal parathyroidectomy (LSPTX) is being explored for suitable candidates [3]. This case report presents a MEN1 patient with PHPT, who underwent LSPTX. We present the following case in accordance with the PROCESS guideline [4].

## 2 Presentation of case

**Fig. 1:**
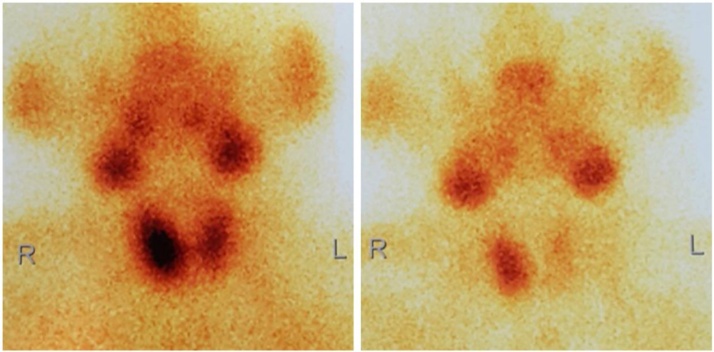
99mTc-MIBI-SPECT/CT suggests hyperparathyroidism of the lower right parathyroid gland.

**Fig. 2:**
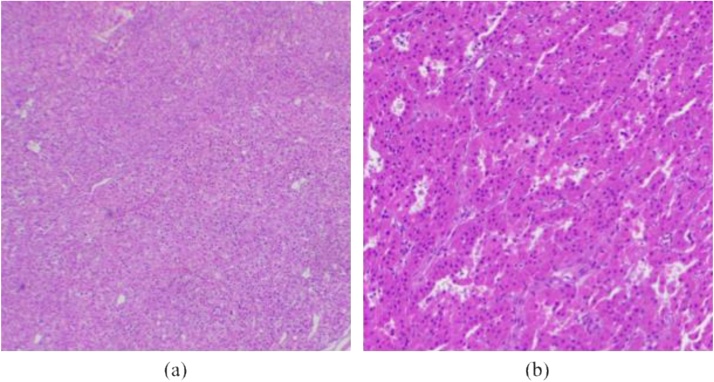
Histopathological analysis showed parathyroid hyperplasia (a) 40× magnification, (b) 100× magnification.

A 28-year-old female was referred to the Urology Department of Dr. Cipto Mangunkusumo Hospital due to recurrent nephrolithiasis for the last five years. Laboratory test showed elevated parathyroid hormone (PTH) and serum calcium level, which were 36.2 pmol/L (normal, 1.6–6.9 pmol/L) and 3.1 mmol/L (normal, 2.2–2.54 mmol/L), respectively. Then she was consulted to the Surgical Oncology Subdivision for further diagnosis and treatment. The patient did not report any other symptoms, and family history was denied. On physical examination, there was no mass palpable in the neck. Ultrasound of the neck did not find any abnormality while Tc-99m MIBI-SPECT/CT showed enhancement at the projection of the lower right parathyroid gland (Fig. 1). Subsequently, parathyroidectomy of the lower right parathyroid gland was performed by the attending surgeon. However, intraoperative parathyroid hormone (IOPTH) measurement was not carried out due to lack of facility. Postoperative histopathology findings were suggestive of parathyroid hyperplasia (Fig. 2).

**Fig. 3:**
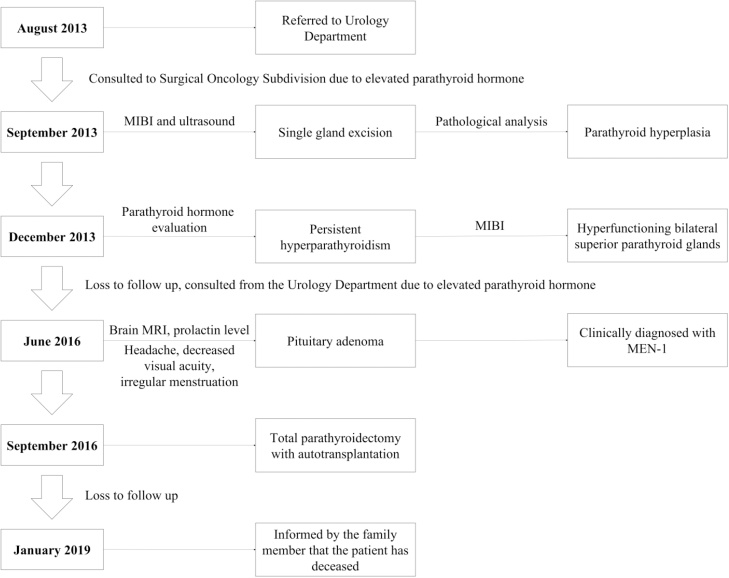
Timeline image of the patient’s history.

Upon follow-up, her PTH level was still elevated (11.2 pmol/L) and 99mTc-MIBI-SPECT/CT showed hyperfunctioning bilateral superior parathyroid glands. Another operation was planned but the patient was lost to follow up. Two years later, she returned to the outpatient clinic after being consulted from the Urology Department. At this time, she complained of irregular menses, frequent headache, and declining visual activity. Further work-up revealed elevated prolactin level (1.80 nmol/L) and a lesion suggestive of pituitary adenoma on her brain MRI. Consequently, she was clinically diagnosed with MEN1, and total parathyroidectomy was performed a few months later. At present, unfortunately, she is deceased due to renal related complications and sepsis (Fig. 3).

## 3 Discussion

Endocrine organs that are classically affected in MEN1 are parathyroid, pituitary, and pancreas. The parathyroid are the most frequently involved (80–98%) with manifestation of symptoms peaking in the third decade of life for women and in the fourth decade for men. MEN1 can be diagnosed either genetically or clinically. Clinical diagnosis can be made if the patient has tumors in two of the three classical endocrine organs or tumor in one organ and a family history of MEN1 [1,5].

Surgery is the main therapy for PHPT in MEN1, and the goal is to achieve normocalcemia while avoiding permanent hypoparathyroidism [1,6]. However, due to its pathogenesis, inadvertently, the remaining normal gland will become abnormal [2,5]. Therefore, the surgery must be planned carefully because higher risk of complications entails repeat surgeries [1,2,5,6]. There are three surgical approaches that have been described but only two are widely practiced, which are SPTX (resection of 3–3.5 glands) and TPTX (resection of 4 glands followed by autotransplantation) [2,6]. The third approach, LSPTX (resection of <3 glands) was deemed inappropriate because of higher risk of persistence (biochemical evidence of hyperparathyroidism ≤6 months after parathyroidectomy) and recurrence (biochemical evidence of hyperparathyroidism >6 months after parathyroidectomy) [1,7]. However, a recent study showed contradicting results. In that study, LSPTX was performed in the form of unilateral clearance (removal of parathyroid glands and cervical thymus on the same side of the neck) for patients with ≥2 concordant preoperative imaging (ultrasound, sestamibi, or 4-dimensional CT) [3].

A systematic search of the literature was conducted on Cochrane library, PubMed, and Scopus to further elucidate the issue. Search strings were constructed using terms under the medical subject headings for MEN1, PHPT, SPTX, TPTX, LSPTX, persistent hyperparathyroidism, and recurrent hyperparathyroidism. Studies comparing SPTX and/or TPTX to LSPTX were included. Studies that did not describe the preoperative imaging findings, non-English studies, and studies that are already included in a systematic review or meta-analysis were excluded. Five studies were eligible for review [3,[8], [9], [10], [11]]. The findings of these studies are summarized in Table 1.

Only two studies reported low risk of recurrence after LSPTX, albeit the small sample size and shorter follow-up [3,8]. However, it is important to note that the other studies did not perform LSPTX based on ≥2 concordant preoperative imaging findings [[9], [10], [11]]. If the same criterion was to be applied, Montenegro et al. mentioned that only 23% of patients who underwent LSPTX were truly eligible [9]. Additionally, thymectomy was not uniformly performed and as such will increase the risk of recurrence or persistence due to the presence of ectopic or supernumerary glands [6,10,11].

Measurement IOPTH also was not uniformly carried out in these studies [3,[8], [9], [10], [11]]. While its use for single gland disease or non-familial PHPT has been established, in MEN1 patients it is less clear. Persistent hyperparathyroidism might still occur even when PTH decreased ≥90% [9,12]. Nevertheless, it is particularly useful, especially if there is a decrease <50%, which prompt suspicion of undetected abnormal glands on preoperative localization studies [9,13].

In our case, the diagnosis of MEN1 was made clinically after the initial parathyroidectomy. The delay in diagnosis of an index case is not uncommon, and mainly attributed to the lack of acknowledgement of MEN1 syndrome and communication between medical doctors [14]. If the diagnosis was made preoperatively, the approach would have been bilateral neck exploration because of the discordant preoperative localization studies. Furthermore, IOPTH measurement, which is now available in our facility, can aid in determining whether the patient has been cured biochemically.

## 4 Conclusion

Currently, SPTX and TPTX are still the preferred approach for PHPT in MEN1. LSPTX might be appropriate in a subset of MEN1 patients, however, further studies are needed due to the lack of evidence on the risk of persistent and/or recurrent hyperparathyroidism.

## Declaration of Competing Interest

None.

## Funding

None.

## Ethical approval

Ethical clearance is exempted for case reports.

FMUI-RSCM Ethical Committee.

## Consent

Consent has been given by the next of kin.

## Author contribution

All authors contributed equally on this work.

## Registration of research studies

Not applicable.

## Provenance and peer review

Not commissioned, externally peer-reviewed.
